# Effect of Regular Training on Platelet Function in Untrained Thoroughbreds

**DOI:** 10.3390/ani14030414

**Published:** 2024-01-27

**Authors:** Arianna Miglio, Emanuela Falcinelli, Katia Cappelli, Samanta Mecocci, Anna Maria Mezzasoma, Maria Teresa Antognoni, Paolo Gresele

**Affiliations:** 1 Department of Veterinary Medicine, University of Perugia, Via San Costanzo 4, 06126 Perugia, Italy; katia.cappelli@unipg.it (K.C.); samanta.mecocci@unipg.it (S.M.); maria.antognoni@unipg.it (M.T.A.); 2 Department of Medicine and Surgery, Section of Internal and Cardiovascular Medicine, University of Perugia, 06126 Perugia, Italy; emanuelafalcinelli@gmail.com (E.F.); annamezzasoma@gmail.com (A.M.M.); paolo.gresele@unipg.it (P.G.)

**Keywords:** coagulation, platelet aggregation, equine, first long-term training, Thoroughbred racehorse

## Abstract

**Simple Summary:**

Hemostatic changes have been demonstrated after exercise in the horse but data are inconclusive. The aim of this study was to investigate platelet activation in young untrained Thoroughbreds in the first training period in order to improve knowledge on this topic. The study included twenty-nine clinically healthy, never-trained, 2-year-old Thoroughbreds. They were followed during their incremental sprint exercise schedule. Blood collection was obtained once a month for five times. Platelet aggregation was measured using a light transmission aggregometer. Platelet function was evaluated using the Platelet Function Analyzer (PFA-100^®^). Nitrite-nitrate (NOx) plasma concentrations were measured to assess in vivo nitric oxide bioavailability. Platelet activation was also investigated through gene expression analyses (selectin P-SELP, ectonucleotidase CD39-ENTPD1, prostaglandin I2 synthase-PTGIS, endothelial nitric oxide synthase 3-NOS3). Significant modifications were identified compared with the beginning of training, with an increase in platelet aggregation and a shorter closure time of PFA-100^®^ that tended to return to its baseline at the end of training. NOx concentrations in plasma significantly increased after 30 days of the training program compared with the baseline. The first long-term training period seems to induce platelet hyperactivity after 30 days in never-trained Thoroughbreds.

**Abstract:**

Training has a significant effect on the physiology of blood coagulation in humans and in horses. Several hemostatic changes have been reported after exercise in the horse but data available are inconclusive. The aim of this study was to investigate platelet activation and primary platelet-related hemostasis modifications in young never-trained Thoroughbreds in the first incremental training period in order to improve knowledge on this topic. Twenty-nine clinically healthy, untrained, 2-year-old Thoroughbred racehorses were followed during their incremental 4-month sprint exercise training. Blood collection was performed once a month, five times in total (T-30, T0, T30, T60, and T90). Platelet aggregation was measured by light transmission aggregometry in response to various agonists: adenosine diphosphate (ADP), collagen, and calcium ionophore A23187. Platelet function was evaluated using a platelet function analyzer (PFA-100^®^) using collagen/ADP and collagen/adrenaline cartridges. Nitrite-nitrate (NOx) plasma concentrations were measured via a colorimetric assay to assess in vivo nitric oxide bioavailability. Platelet activation was also investigated through gene expression analyses (selectin P-*SELP*, ectonucleotidase CD39-*ENTPD1*, prostaglandin I2 synthase-*PTGIS*, endothelial nitric oxide synthase 3-*NOS3*). Differences among the time points were analyzed and mean ± SEM were calculated. Significant modifications were identified compared with T-30, with an increase in platelet aggregation (collagen:32.6 ± 4.8 vs. 21.6 ± 4.9%; ADP: 35.5 ± 2.0 vs. 24.5 ± 3.1%; A23187: 30 ± 4.7 vs. 23.8 ± 4%) and a shorter closure time of C-ADP cartridges (75.6 ± 4.4 vs. 87.7 ± 3.4 s) that tended to return to the baseline value at T90. NOx concentrations in plasma significantly increased after 30 days of the training program compared with the baseline. The first long-term training period seems to induce platelet hyperactivity after 30 days in never-trained Thoroughbreds. Regular physical training reduces the negative effects of acute efforts on platelet activation.

## 1. Introduction

In horses, cardiovascular disease is less common than in humans, dogs, or cats. However, because horses often perform athletic efforts, the cardiovascular condition of the animal is important. Physical exercise triggers important mechanisms related to the exercise benefits for health improvement and protects against cardiovascular risk. As platelets play a key role in arterial thromboembolic disease, the effect of exercise on platelet function is of special interest. Intense physical training has a strong effect on blood hemostasis both in humans and in horses [1,2,3,4,5,6]. In horses, exercise and training are known to have considerable effects on the mechanisms of the hemostatic system involving platelet activity. Hemostatic changes have been reported after exercise in horses but with contrasting data. Moreover, many studies have been performed only on the occasion of acute exercise [3,5,6,7,8,9]. Most of the studies assessing the influence of training on platelet aggregation in Thoroughbred racehorses were focused on the acute effects of one single strenuous exercise, which seems to transiently inhibit platelet reactivity, possibly due to a protective activation of the endothelium with the production of nitric oxide in response to physical exercise [3,9]. Nevertheless, the results of these studies are conflicting due to differences in the anticoagulants used for blood sampling, analytical methods used, and the effect of different training programs (exercise intensity, duration, and fitness status) [2,10,11,12]. Depending on the intensity, duration, and type of physical exercise, the equine metabolism also has to adapt to endocrine system requirements. For example, different fitness levels influence cortisol release in endurance and racehorses [13], and in humans, a negative relationship between the plasma concentration of cortisol and platelet reactivity to arachidonic acid and ADP has recently been shown [14]. Moreover, it was also recently shown that proteins involved in the regulation of platelet function, such as matrix metalloproteinase 2 (MMP-2) [15], were shown to change in racehorses at different levels of training [16]. 

The physiological regulation of platelet reactivity is tightly controlled by balancing prothrombotic and antithrombotic signals. In humans, nitric oxide (NO), a gaseous signaling molecule involved in many physiological functions, is one of the potent inhibitors of platelet activation. Its antithrombotic effect is the consequence of the blunting of human platelet adhesion to the endothelium and aggregation [17,18,19]. Several studies suggested that training and exercise increase NO generation and reduce NO deactivation, thus increasing serum/plasma NO levels in humans [20].

The potential effect of NO on platelet activation pathways has not been explored in detail in horses and no studies have been carried out on NO regulation of platelet function in racehorses during the training period. 

Poor performance is a common, multifactorial syndrome in racehorses, usually associated with subclinical disorders. One of the most common medical causes is exercise-induced pulmonary hemorrhage (EIPH) [21]. This common bleeding disorder can occur in the lungs of Thoroughbred and Standardbred horses during and after strenuous exercise [22,23] and adversely affects the health, well-being, and athletic ability of racehorses. The knowledge of any platelet alterations that predispose to the onset of EIPH is poor.

The aim of our study was to investigate changes in platelet activation and NO activity, as well as in the expression of some genes related to hemostasis and inflammation (selectin P-SELP, ectonucleotidase triphosphate diphosphohydrolase-1 CD39-ENTPD1, prostaglandin I2 synthase-PTGIS, and nitric oxide synthase 3-NOS3) [24,25,26,27,28,29], in young, never-trained Thoroughbreds during the first incremental exercise period, in order to deeply understand the dynamics of the coagulation system in this athletic horse. Monitoring of blood hemostasis could provide useful information about the fitness level of the animals during exercise and competition and might contribute to the development of more adequate training programs. Moreover, it could be useful in detecting particular modifications of the hemostatic balance predisposing the sport horses to bleeding manifestations. 

## 2. Material and Methods

### 2.1. Animals

Twenty-nine Thoroughbreds (17 males and 12 females; two years old, weight 450–550 kg, height 165–168 cm) were enrolled (the same animals were included in our previous studies [30,31]). Horses were clinically healthy (no previous coagulative abnormalities) based on heart examination, thorax auscultation, and rectal temperature. Hemato-biochemical and hemostatic analyses were within reference ranges. No pharmacological treatment was assumed prior to or during the study.

Horses were housed in an approved facility located in Central Italy (Rome) in accordance with protocols prescribed by the Institutional Animal Care and Use Committee. All horses were managed with the same feeding and housing schedule. These animals were never trained for flat racing (canter and gallop) before the beginning of the study. Each horse followed a feeding program to maintain health, performance, and well-being during training. Water was available ad libitum. 

Horses were trained with the same training schedule (Table 1) for a 4-month period (from April to July 2018, from Monday to Saturday, with rest on Sunday). Clinical evaluation was conducted and blood samples were taken at rest: 30 days before the beginning of the official training program when horses had never trained, prior to when they were housed together (T-30); on the day of the beginning of the training program, which included canter and gallop (T0); and at 30 days (T30), 60 days (T60), and 90 days (T90) after the incremental increase in the training program, in order to assess whether or not animals had adapted to the exercise (Table 1). Each animal had one or more races after the end of the experimental period. No horses showed poor-performance syndrome during the study period or the races. 

### 2.2. Sample Collection

Blood sampling activity was performed once a month, from March 2018 to July 2018, at 6:30 a.m., at rest, before training and feeding. In March (T-30, untrained horses), horses were housed together and began to have the same environmental conditions of nutrition and mild exercise. April (T0, horses starting gallop) was the first month of the beginning of training simulating competitions (gallop; samples were taken at rest on the first day of training). From April to July, training was incremental (T30, T60, T60, T90; horses under incremental training program). Blood samples were collected directly into Vacutainer tubes via venipuncture of the jugular vein, avoiding unnecessary manipulation that could induce activation of coagulation. Blood samples were collected into Vacutainer tubes (Terumo Corporation, BD brand; Tokyo, Japan) with K3-ethylenediamine tetra acetic acid-EDTA or with 3.8% sodium citrate. K3-EDTA tubes were used to evaluate the expression of target genes in an RT-qPCR system. Sodium citrate tubes were used to evaluate platelet function and for nitrite-nitrate (NOx) assay. One sodium citrate blood tube was centrifuged at 1500× *g* for 15 min to obtain citrated plasma samples within 15 min following collection. All samples were immediately transported to the coagulation laboratory and were analyzed within 3 h of blood collection.

### 2.3. Light Transmission Aggregometry (LTA)

To study platelet aggregation, platelet-rich plasma (PRP) was obtained by centrifugation of citrated blood at 120 g for 15 min at room temperature. The upper 2/3 of PRP was carefully removed using a plastic pipette and transferred into plastic tubes. Platelet-poor plasma (PPP) was prepared by further centrifugation of the remaining blood at 4000× *g* for 10 min [32]. 

Platelet aggregation was carried out using an optical aggregometer (APACT-4, Helena Biosciences, London, UK), as previously described [24]. Agonists used were adenosine diphosphate (ADP; 1, 5, and 10 µM), collagen (4 µg/mL), and calcium ionophore A23187 (5 µM) [32,33].

Results are expressed as maximal amplitude of aggregation measured within 5 min of stimulation.

### 2.4. Platelet Function Analyzer

A platelet function analyzer (PFA-100^®^, Siemens Healthcare, Malvern, PA, USA) is a point-of-care platelet function analyzer that evaluates platelet function in a small volume of whole blood in high shear-stress conditions and in a semiautomatic system [34,35]. Recent studies have reported the use of the PFA-100^®^ for the evaluation of primary hemostasis in horses [29]. The device simulates the process of primary hemostasis by triggering platelet adhesion and hemostatic plug formation under high shear flow conditions. Citrated whole blood is aspirated through an aperture through a collagen membrane coated with platelet agonists (collagen and adenosine diphosphate (C-ADP) or collagen and epinephrine (C-EPI)). This design mimics in vivo endothelial damage and platelet plug formation [35]. Time until plug formation was measured in seconds and was defined as closure time [36]. The test was performed according to the manufacturer’s instructions.

### 2.5. Nitrite-Nitrate (NOx)

As Nitric oxide (NO) has a very short half-life, the plasma concentrations of oxidized NO metabolites (NOx) nitrate (NO_3_^−^) and nitrite (NO_2_^−^) are frequently used to assess NO bioavailability in vivo. Nitrite-nitrate (NOx) plasma concentrations, an indirect measurement of NO generation in biological fluids, were measured using a colorimetric assay kit (R&D system Minneapolis, MN, USA), according to the manufacturer’s instructions, as previously described [37,38].

### 2.6. RT-qPCR Analyses

We examined gene expression profiles in peripheral blood leukocytes of horses during the training program. mRNA expression values were normalized with three reference genes (*SDHA*, *B2M*, and *HPRT*). Total RNA was extracted from buffy coat recovered from 10 mL of total blood as previously described [31,39]. The resulting pellets were resuspended in 2 mL of TriZol reagent at room temperature (Thermo Fisher Scientific, Waltham, MA, USA) and RNA was isolated using a TriZol Plus RNA purification kit (Thermo Fisher Scientific, Waltham, MA, USA), as previously described, from T-30, T0, T30, and T90 samples. The quantity of RNA was checked using a NanoDrop2000 spectrophotometer (Thermo Fisher Scientific, Waltham, MA, USA) and quality was checked via electrophoresis in a denaturing 1.2% agarose gel. 

One microgram (1 µg) of total RNA of each sample was reverse transcribed using the SuperScript^®^VILO IV^TM^ Master Mix (Thermo Fisher Scientific, Waltham, MA, USA) according to the manufacturer’s recommendations. Primers for reference genes Succinate dehydrogenase complex, subunit A (*SDHA*), and Beta-2 microglobulin (*B2M*), for optimal housekeeping for blood cells in horses, as well as *IL*-*1*β genes of interest, were previously determined [40], while for genes of interest *SELP*, *ENTPD1*, *PTGIS*, and *NOS3*, primers were designed on available sequences using the Primer-BLAST software (https://www.ncbi.nlm.nih.gov/tools/primer-blast/ accessed on 3 January 2021), trying to place them in different exons or at exon–exon junctions to avoid biases due to genomic DNA amplification. Primer sequences and accession numbers for tested genes are listed in Table 2. The relative normalized expression was calculated using the 2^−∆∆Ct^ method, using T0 as control, with CFX Maestro software (ver. 4.1 BioRad, Hercules, CA, USA). 

### 2.7. Statistical Analysis

All results are expressed as mean ± standard error (SEM). Differences among time points were analyzed by the one-way repeated measures ANOVA. A *p* value < 0.05 was considered statistically significant.

RT-qPCR samples were divided into groups (T-30, T0, T30, and T90) and the Shapiro–Wilk test was used to determine the likelihood that the expression values of the samples in a biological group were obtained from a normally distributed population. Then, modifications in the relative gene expression between groups were evaluated using one-way ANOVA. Compact letter displays (CLDs) were used to highlight significance (*p* < 0.05). Data are expressed as means of fold change with the standard error using CFX maestro software (ver. 4.1 BioRad, Hercules, CA, USA).

## 3. Results

### 3.1. Effect of Training on Platelet Activation

**Figure 1:**
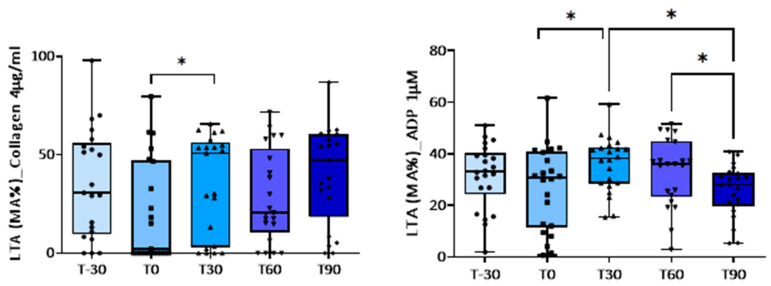
Light transmission aggregometry, expressed as maximal amplitude (MA%), induced by collagen (4 µg/mL) and ADP (1 µM) at baseline and during the training program. * *p* < 0.05.

**Figure 2:**
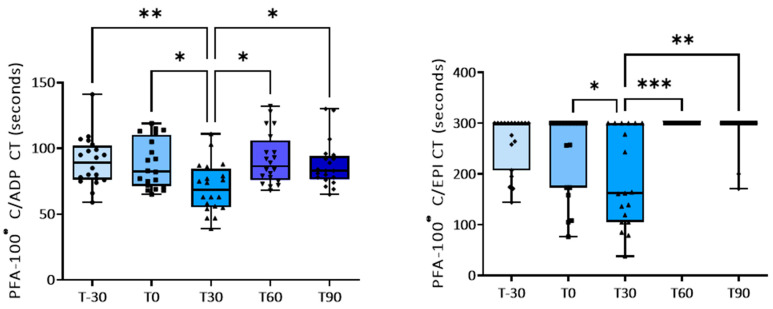
PFA-100^®^ closure time (sec) with collagen/ADP (C/ADP) and collagen/EPI (C/EPI) cartridges at baseline and during the training program. * *p* < 0.05; ** *p* < 0.01; *** *p* < 0.001.

Platelet aggregation induced by low concentrations of ADP, by collagen, and by A23187 increased significantly after 30 or 60 days of the training program compared with baseline (T0) (Table 3 and Figure 1). Exercise had no significant effect on platelet responses to higher concentrations of ADP (Table 3). Concomitantly, a shorter closure time of the C-ADP cartridge (70.0 ± 4.2 vs. 89.8 ± 4.1 s) and of the C-EPI cartridge (190.0 ± 21 vs. 242.4 ± 21 s) with the PFA-100^®^analyzer was observed at T30 compared to baseline (Table 4 and Figure 2), confirming platelet hyperreactivity (Table 4). Most of these parameters tended to return to the basal values at the end of training period.

### 3.2. NO Metabolism

**Figure 3:**
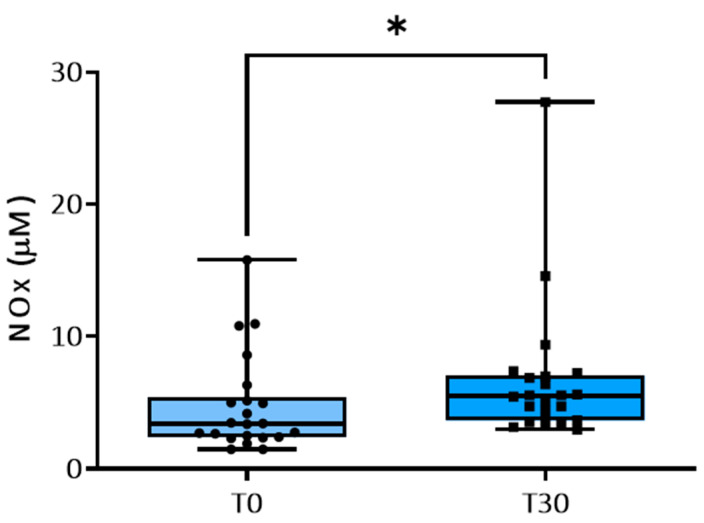
Plasma concentrations (uM) of nitrite and nitrate (NOx) at baseline and at 30 days of training. * *p* < 0.05.

Compared to the values measured at T0, physical training significantly increased NOx concentrations in plasma at T30 (Figure 3). 

### 3.3. RT-qPCR Analyses

**Figure 4:**
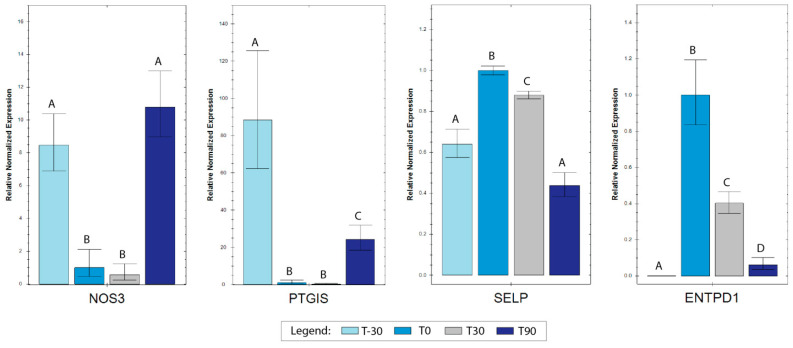
Histograms representing the relative normalized expression values for the investigated genes at T-30, T0, T30, and T90. Data are expressed as ΔΔCq ± 1 standard error, setting as T0 as control group. Statistical significance (*p* < 0.05) was tested using pairwise comparisons for all the training times through compact letter displays (CLDs): A,B,C,D: different letters within each box indicate statistical significance.

Reference genes showed high stability, with an M value below the accepted threshold [41]. Differences in gene expression for target genes were assessed setting T0 as the control time and carrying out pairwise comparisons between all the different sampling times. All the tested genes (*SELP*, *ENTPD1*, *PTGIS*, and *NOS3*) were found to be modulated (Figure 4), with *PTGIS* and *NOS3* showing a decrease in T0 and T30 compared to T-30 and both increasing again in T90. Indeed, *NOS3* returned to the same level as that of T-30, while *PTGIS,* although up-regulated, maintained lower expression levels with respect to T-30. For *ENTPD1* and *SELP*, an opposite trend with respect to the first two genes was found. *ENTPD1* showed a marked increase in T0 compared to T-30 and a progressive reduction in T30 and T90, although the expression levels for these two time points were kept higher compared to in T-30. *SELP* was characterized by an increased expression in T0 compared to T-30, which decreased again in T30 and further in T90, returning to the initial expression levels for the last time point.

## 4. Discussion

Our study shows that the first long-term training period in untrained Thoroughbreds induces significant changes in platelet activation, which become apparent after 30 days. In fact, a transiently increased platelet aggregation induced by collagen, a low dose of ADP, and calcium ionophore A23187 associated with a shortening of the PFA-100^®^ closure time were observed. These effects of training on platelet activation tended to disappear after 90 days, indicating an adaptation of the primary hemostasis system to long-term exercise training. 

The few studies that have assessed the influence of training on platelet aggregation in thoroughbred racehorses were focused on the acute effects of one single strenuous exercise, and results are conflicting due to differences in the anticoagulants used for blood sampling, analytical methods used, and the effect of different training programs (exercise intensity, duration, and fitness status) [2,10,11,42]. Here we show for the first time that young Thoroughbreds during their first long incremental training season display an enhanced platelet activation. We demonstrate an increased aggregation of platelets after 30 days of training with a subsequent tendency to return to basal concentrations with the continuation of training. These results suggest the development of a condition of transient platelet hyperactivity soon after the start of intensive training which tends to subside with the habituation to physical exercise. Our data are in accordance with data reported in sportsmen undergoing strenuous long training [10], but they differ from previous studies in Thoroughbreds [43] and in jumping horses evaluated during 5 weeks of training [2], probably due to the different analytical methods used and the fitness status of the animals. To our knowledge, most of the published studies evaluating platelet aggregation in horses used impedance aggregometry [2,43,44,45,46] and only rarely light transmission aggregometry [47,48], in some cases with poorly validated devices. Light transmission aggregometry is considered the gold-standard test for the assessment of platelet function and for the monitoring of bleeding risk [35], while impedance aggregometry does not appear to have sufficient sensitivity or specificity to detect platelet function alterations. Finally, almost all previous studies used a single agonist, e.g., ADP [2,43,45,46] or collagen [49], while we evaluated various pathways of platelet activation by using a panel of agonists and, for the first time in the horse, using the calcium ionophore A23187, which, as well as ADP and collagen, seems to be active on platelets in this species. More studies are needed to standardize platelet agonists for horse platelet studies and to identify reference values for the gold-standard light transmission aggregometry. 

Over the past decades, assessment of platelet function in vitro has been used in human medicine to study primary hemostasis. The PFA-100^®^ analyzer evaluates platelet function in vitro in whole blood by simulating platelet adhesion, secretion, and aggregation after a vascular injury. Moreover, PFA-100^®^, differently from LTA, is a test that relies on high shear rates. In veterinary medicine this method has been recently shown to be sensitive to and accurate in the diagnosis of platelet dysfunction in pigs, dogs, and horses using species-specific reference values [32]. We analyzed, for the first time, platelet function in young untrained Thoroughbreds during the first long-term training period by using the PFA-100^®^. In our study, a shorter C-ADP and C-EPI closure time at T30 confirmed increased platelet reactivity, which tended then to return to basal concentrations at the subsequent time points. The mean CT identified in Thoroughbreds at T0 was similar to that previously identified (85.1 ± 13.1 s) in normal horses [32].

Our results also show increased plasma concentrations of NOx after 30 days of incremental training simulating competitions. Previous human and animal studies have reported increased plasma concentrations of the NO degradation products nitrite and nitrate after acute exercise [50]. As an increased generation of NO might be counterbalanced by its inactivation, for example, by reactive oxygen species (ROS), measured concentrations of NO metabolites do not always indicate biologically active NO. In fact, during physical activity, muscles spasm, accompanied by damage to the muscle fibers, which initiates an inflammatory response to exercise and pro-inflammatory cytokines, such as IL-1β, and activates neutrophils and nicotinamide adenine dinucleotide phosphate (NADPH) oxidase enzymes, which in turn enhance the production of ROS and free radicals. Our results suggest that studies on NO bioavailability in horses during exercise training are warranted. Unfortunately, we did not evaluate ROS and free radicals in the horses included in this study. However, a significantly decreased ROS production by peripheral blood mononuclear cell was recently observed in well-trained compared to untrained racehorses [51]. 

We also assessed the expression of some genes known to be related to hemostasis and inflammation in humans (*SELP*-selectin P, *ENTPD1*-ectonucleotidase CD39, *PTGIS*-prostaglandin I2 synthase, *NOS3*-endothelial nitric oxide synthase 3) to deeply investigate the observed exercise-induced changes in platelet activation.

*SELP* encodes a 140 kDa protein (P-selectin) constitutively expressed by platelets and endothelial cells, essential for the interactions between blood cells and the endothelium and involved in the inflammation response. Our data show that after 30 days of exercise, *SELP* expression increased return to baseline values after long-term exercise (T90), suggesting adaptation to exercise training. Endothelium-derived inhibitory mechanisms play an important role in regulation of platelet activation. These mainly include nitric oxide (NO), prostacyclin PGI2, and adenosine, which are synthesized by endothelial NO synthases (eNOS), prostacyclin synthase (PTGHS), and CD39/CD73. Vascular laminar shear stress increases during exercise and is associated with a rapid up-regulation of endothelial nitric oxide synthase (eNOS) mRNA and protein expression levels [52]. Previous studies in rats showed that exercise training increased endothelial NO synthesis, suggesting that increased production of endothelial NO constitutes an initial phase in the adaptive response to exercise training [53]. Our results show an increase in eNOS transcription between initial phases of training (T0 and T30) and the end of the monitored training period (T90), whereas eNOS expression was unchanged between T-30 and T90. 

Prostacyclin synthase catalyzes the last step of prostaglandin I2 synthesis, a potent inhibitor of platelet aggregation. Interestingly the gene encoding for prostacyclin synthase, PTGIS, was down-regulated at all time points compared to T-30, suggesting a possible reduced prostaglandin I2-mediated inhibition of platelet aggregation. Meanwhile, there was a partial attempt to increase PTGIS expression at T90 compared to T0 and T30, probably indicating an adaptation to training and an effort to restore homeostasis.

CD39/CD73 constitutes a further pathway that regulates platelet function. The *ENTPD1* gene codes for CD39, which is a protein implicated in the prevention of platelet aggregation by hydrolyzing platelet-activating ADP to AMP. In platelets, adenosine triphosphate (ATP) and adenosine diphosphate (ADP) are stored in dense granules and released upon activation. ATP and ADP interact with specific receptors on platelets (P2Y_1_ and P2Y_12_) to activate and recruit additional platelets to the site of vascular injury to form a thrombus. Within the vasculature, ATP and ADP concentrations are regulated by transmembrane nucleotidases that rapidly degrade ATP and ADP to adenosine, thus diminishing platelet activation and preventing thrombotic events. There is evidence that in an inflammatory environment, the loss of CD39 activity from the endothelium sustains platelet aggregation and thrombogenesis [54]. In our white blood cell samples, an increase in *ENTPD1* for all time periods compared to T-30 was observed, indicating a mechanism facilitating platelet activation by exercise. Indeed, vigorous exercise acutely elevates the expression of CD39 on lymphocytes, promoting the degradation of ATP to adenosine via the CD39/CD73 pathway [55]. However, a reduction in *ENTPD1* expression characterized T30, and even more T90, indicating a gradual return to the basal situation. 

Our study included the assessment of numerous factors and genes involved in platelet function, with the purpose of fully understanding the dynamics of this system in the athletic horse in response to long-term training programs. It seems that during the first exercise season most parameters had the maximum changes after 1 month (T30) of training in never-trained horses, and that an adaptive response to conditioning probably began after two months (T60) of incremental training and continued after three months (T90). This agrees with what was found in previous studies regarding platelet count and clotting parameters [30,56]. As in our study, clotting parameters showed increased activity (decreased prothrombin time and thrombin time, and increased fibrinogen concentrations) 30 days after the beginning of training, but these tended to return to basal concentrations as an adaptation to the training program solely 3 months later, showing a later recovery compared to platelet parameters [30].

The main limitations of this study are the limited number of animals and the lack of a control group not entering training, even if each animal was evaluated 1 month before the training period (T-30) to define the starting point, and the subjective reporting of workloads by trainers. In fact, because it is impossible to have a control group at the same age as untrained Thoroughbreds, we sampled a group of horses that had never been trained before to have a control group that comprised untrained subjects. To also reduce the environmental effects, we waited 30 days after housing, at which time the animals arrived at the same training center, with standardized management and diet, and began light exercise (T-30; T0).

To strengthen our observations, it might be beneficial to delve into a long-term analysis of training effects, understand the interactions of platelets with other cells, and assess their behaviour under different exercise regimens. Investigating the molecular mechanisms through which platelets operate, their impact on other equine cells such as peripheral blood mononuclear cells (PBMCs), and linking the cellular benefits to behavioral or performance outcomes in horses could provide a more holistic view of regular exercise benefits in equine health.

## 5. Conclusions

The first long-term exercise period was found to induce platelet hyperactivity after 30 days of training in never-trained Thoroughbreds. Regular physical training attenuates the negative consequences of acute effort on platelet activation. This effect may be attributed to training status. It seems probable that the platelet adaptive response to conditioning occurs after two months of training in young untrained Thoroughbred horses during the first training season. These results seem to be promising, since they allow us to more deeply understand the dynamics of the hemostatic system in this athletic horse during his first training period, contributing to the development of more adequate training programs. Interestingly, they could also be useful in detecting possible hemostatic alterations predisposing to bleeding disorders in sport horses. 

## Figures and Tables

**Table 1:** Standard daily training program completed by each horse involved in the study. Speeds: walk: 100 m/min, trot: 200 m/min, canter: 350 m/min, and gallop: 1000 m/min (min: minutes).

March (T-30)	April (T0)	May (T30)	June (T60)	July (T90)
Untrained horses	Start of training	Incremental training	Incremental training	Incremental training
15 min Walk	15 min Walk	15 min Walk	15 min Walk	15 min Walk
10 min Trot	10 min Trot	10 min Trot	10 min Trot	10 min Trot
Rest	6 min Canter	6 min Canter	6 min Canter	6 min Canter
10 min Trot	Every Tuesday:	Every Tuesday:	Every Tuesday:	Every Tuesday:
Walk	1 min Gallop	2 min Gallop	3 min Gallop	4 min Gallop

**Table 2:** Genes evaluated in this study and relative primer pairs used.

Gene	Primer Forward	Primer Reverse	Amp. Length	Accession
*SELP*	GCTGACAATCCAGGAAGCCC	CGCTTTGAGCAGTCAAGGGA	146	NM_001081792
*ENTPD1*	TTGAGCCACCAAGACCAGAAG	ATTCTGGGTCAACCCCACAG	125	XM_001500628
*PTGIS*	TTCCTGAGTCCGCAGAAGGA	TCGCTTCCCGTCCTTGTAAA	117	XM_001501166
*NOS3*	TTCGGGAGAGTGAGCTGGTA	CAATCCCGCGCATCAAAGAC	109	XM_001504650
*B2M*	TCCTGCTCGGGCTACTCTC	TGCTGGGTGACGTGAGTAAA	83	NM_001082502
*SDHA*	GCGCGCTTCAGACGATTTAT	CCAGTGCTCCTCAAATGGCT	146	XM_014734954

**Table 3:** Maximal amplitude (MA) of platelet aggregation induced by several agonists at baseline and during the training program. Data are reported as mean ± SEM. Significant differences are expressed with *p* values (*p* < 0.05).

Maximal Amplitude (%)	T-30	T0	T30	T60	T90
Collagen (4 µg/mL)	35.4 ± 6.0	21.0 ± 5.7	35.0 ± 5.0 *p* = 0.05 vs. T0	28.8 ± 5.1	40.4 ± 5.4
ADP (1 µM)	31.5 ± 2.7	26.9 ± 3.4	35.9 ± 2.2 *p =* 0.008 vs. T0; *p =* 0.013 vs. T90	33.1 ± 2.8 *p* = 0.029 vs. T90	25.7 ± 2.1
ADP (5 µM)	58.4 ± 3.7	57.8 ± 1.9	61.0 ± 3.0	60.8 ± 4.6	60.5 ± 3.6
ADP (10 µM)	63.5 ± 2.3	63.3 ± 1.6	64.4 ± 2.2	59.3 ± 2.2	62.3 ± 2.1
A23187 (5 µM)	Not done	24.2 ± 5.1	30.9 ± 5.2	41.8 ± 5.3 *p =* 0.03 vs. T0; *p* = 0.04 vs. T90	22.0 ± 4.9

**Table 4:** PFA-100^®^ closure times (sec) with collagen (C)-ADP and collagen (C)-EPI cartridges at baseline and during the training program. Data are reported as mean ± SEM. Significant differences are expressed with *p* values (*p* < 0.05).

PFA-100^®^ (sec)	T-30	T0	T30	T60	T90
C-ADP	89.8 ± 4.1	88.2 ± 4.1	70.0 ± 4.2 *p* = 0.03 vs. T0; *p* = 0.0021 vs. T-30	92.0 ± 4.4 *p* = 0.011 vs. T30	87.6 ± 3.9 *p* = 0.019 vs. T30
C-EPI	262.0 ± 12.5	242.4 ± 18.0	190.0 ± 21.0 *p* = 0.045 vs. T0	300 ± 0.0 *p* = 0.0006 vs. T30	287.0 ± 8.3 *p* = 0.0021 vs. T30

## Data Availability

Data are contained within the article.
